# Intraoperative CT and cone-beam CT imaging for minimally invasive evacuation of spontaneous intracerebral hemorrhage

**DOI:** 10.1007/s00701-020-04284-y

**Published:** 2020-03-19

**Authors:** Nils Hecht, Marcus Czabanka, Paul Kendlbacher, Julia-Helene Raff, Georg Bohner, Peter Vajkoczy

**Affiliations:** 1 Department of Neurosurgery and Center for Stroke Research Berlin (CSB), Charité - Universitätsmedizin Berlin, Charitéplatz 1, 10117 Berlin, Germany; 2 Department of Neuroradiology, Charité - Universitätsmedizin Berlin, Berlin, Germany

**Keywords:** Minimally invasive surgery, Intracerebral hemorrhage, Stroke, Intraoperative imaging, Cone-beam CT, Intraoperative CT, Image guidance

## Abstract

**Background:**

Minimally invasive surgery (MIS) for evacuation of spontaneous intracerebral hemorrhage (ICH) has shown promise but there remains a need for intraoperative performance assessment considering the wide range of evacuation effectiveness. In this feasibility study, we analyzed the benefit of intraoperative 3-dimensional imaging during navigated endoscopy-assisted ICH evacuation by mechanical clot fragmentation and aspiration.

**Methods:**

18 patients with superficial or deep supratentorial ICH underwent MIS for clot evacuation followed by intraoperative computerized tomography (iCT) or cone-beam CT (CBCT) imaging. Eligibility for MIS required (a) availability of intraoperative iCT or CBCT, (b) spontaneous lobar or deep ICH without vascular pathology, (c) a stable ICH volume (20–90 ml), (d) a reduced level of consciousness (GCS 5–14), and (e) a premorbid mRS ≤ 1. Demographic, clinical, and radiographic patient data were analyzed by two independent observers.

**Results:**

Nine female and 9 male patients with a median age of 76 years (42–85) presented with an ICH score of 3 (1–4), GCS of 10 (5–14) and ICH volume of 54 ± 26 ml. Clot fragmentation and aspiration was feasible in all cases and intraoperative imaging determined an overall evacuation rate of 80 ± 19% (residual hematoma volume: 13 ± 17 ml; *p* < 0.0001 vs. Pre-OP). Based on the intraoperative imaging results, 1/3rd of all patients underwent an immediate re-aspiration attempt. No patient experienced hemorrhagic complications or required conversion to open craniotomy. However, routine postoperative CT imaging revealed early hematoma re-expansion with an adjusted evacuation rate of 59 ± 30% (residual hematoma volume: 26 ± 37 ml; *p* < 0.001 vs. Pre-OP).

**Conclusions:**

Routine utilization of iCT or CBCT imaging in MIS for ICH permits direct surgical performance assessment and the chance for immediate re-aspiration, which may optimize targeting of an ideal residual hematoma volume and reduce secondary revision rates.

**Electronic supplementary material:**

The online version of this article (10.1007/s00701-020-04284-y) contains supplementary material, which is available to authorized users.

## Introduction

Intracerebral hemorrhage (ICH) accounts for up to 15% of all strokes and affects more than 2 million people annually [32]. Of all stroke subtypes, ICH is associated with the poorest prognosis: Of the 50% surviving the initial hemorrhage and acute hospitalization, more than 2/3rd remain functionally dependent. The main reason for this is that ICH remains the least treatable of all stroke types and apart from stroke unit care, no treatment has unequivocally shown clinical effectiveness [12]. This lack of effectiveness of any specific treatment has led to skepticism about the potential for surgical ICH evacuation, because it has been speculated that the failure of conventional surgery to benefit these patients [26, 27] is related to the morbidity of the surgical approach. Consequently, a number of MIS techniques, such as stereotactic thrombolysis [18, 29], hematoma evacuation through a tubular retractor [24], and navigated, endoscopic ICH evacuation with mechanical clot fragmentation and aspiration [21, 37], have been developed and meanwhile suggest superiority versus conventional surgery and medical management [33]. Nevertheless, the surgical performance of MIS for ICH remains a matter of debate and this is important, because recent evidence has demonstrated that hematoma reduction below a certain threshold is required to provide the highest chance of reaching a favorable long-term outcome [3].

For neurosurgical performance assessment, intraoperative imaging such as MRI [7, 10, 35], angiography [13, 22], and computerized tomography (iCT) [20] or cone-beam CT (CBCT) [11] have been evidenced to increase patient safety and provide a measure of immediate quality control. For cranial imaging in MIS for ICH, the simple and fast use of iCT and CBCT appears particularly attractive but the benefit of using this technology in that setting has not yet been investigated. In the present study, we therefore analyzed the benefit of navigated, endoscopy-assisted ICH evacuation by mechanical clot fragmentation and aspiration in combination with intraoperative CT or CBCT imaging.

## Methods and materials

### Study design

The study was approved by the local ethics committee of the Charité University Hospital in Berlin, Germany (EA1/223/19) and performed in compliance with the Health Insurance Probability and Accountability Act regulations. Informed consent was waived due to the retrospective nature of the study. Between July 2016 and July 2019, 18 consecutive patients that underwent navigated, endoscopy-assisted evacuation of spontaneous intracerebral hemorrhage using a mechanical fragmentation and aspiration device (Penumbra Inc., Alameda, CA, USA) were identified. Demographic, clinical and radiographic patient data with focus on surgical workflow, pre-, intra-, and postoperative hematoma volumes, procedure-related complications, and outcome measured as the level of consciousness according to the Glasgow Coma Scale (GCS) score before and at the time of discharge and the 30-day mortality were collected and analyzed by two clinicians who were not directly involved in the patients’ care (PK, JHR).

### Patient management

All patients were treated according to the guidelines of the German societies of Neurology and Neurosurgery. The indication for minimally invasive hematoma evacuation was based on previous findings [16, 18, 27, 33] under consideration of the following aspects:

- Availability of intraoperative CT or CBCT imaging.
- Supratentorial location without vascular pathology (excluded by computerized tomography angiography (CTA) and/or digital subtraction angiography (DSA)).
- Hematoma volume 20–90 ml.
- Reduced level of consciousness with a Glasgow Coma Scale (GCS) score 5–14.
- Premorbid modified Rankin Scale (mRS) score ≤ 1.
- Stable control CT after 6 h without spot sign and surgery ideally within 72 h.

Further information on patient management and intraoperative imaging can be found in the online supplement. In addition to intraoperative imaging, a routine CT scan was performed on postoperative day (POD) 1. Additional imaging was performed depending on the individual clinical course.

**Fig. 1:**
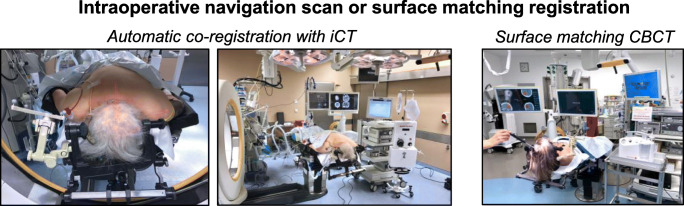
Positioning and navigation registration. The left panels show the iCT setup for automatic patient/image co-registration in a case without preoperative registration CT (patient 1): For iCT scanning, the head is fixed in a radiolucent clamp that holds the navigation tracking device and the patient is rotated into the iCT gantry (left). For surgery, the OR table is rotated into surgery position (center). The right panel shows the CBCT setup for surface matching registration in a case with preoperative registration CT (patient 15)

**Fig. 2:**
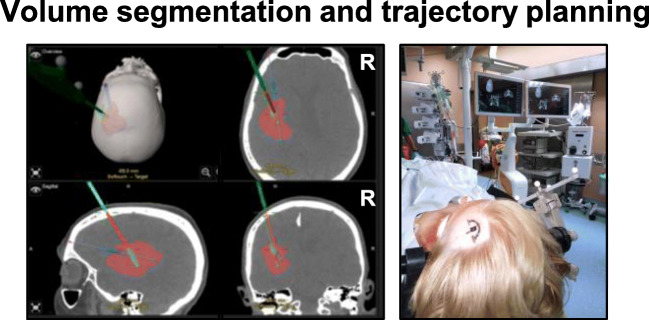
Segmentation and planning. The segmentation of the hematoma volume with planning of the trajectory is shown for patient 2 (left panel). Hematoma segmentation (red) and trajectory planning according to the longest hematoma axis (blue trajectory) were performed directly in the OR. After surface matching of the preoperative image data set the left frontal entry point and skin incision are marked accordingly (right panel)

**Fig. 3:**
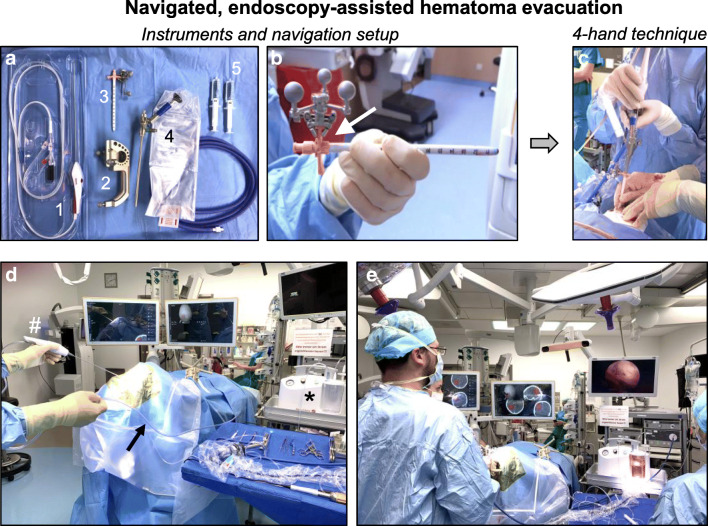
Instruments and hematoma evacuation. **a** Instruments required for navigated, endoscopy-assisted hematoma aspiration: (1) Fragmentation and aspiration device, (2) navigation tool for referencing of the endoscope sheath, (3) endoscope sheath with navigation tracking device, (4) endoscope, (5) irrigation. **b** Endoscope sheath with tracking device clamped to the distal end of the sheath. **c** 4-hand technique for hematoma evacuation with one surgeon performing endoscopy-assisted aspiration and the second surgeon guiding the navigated sheath and endoscope. **d** Setup using the second-generation fragmentation and aspiration device with longer aspiration tubing (arrow) that connects the wand (number sign) to the aspiration pump (asterisk). **e** Positioning of the surgical team, aspiration pump, endoscopy system, and image guidance

**Fig. 4:**
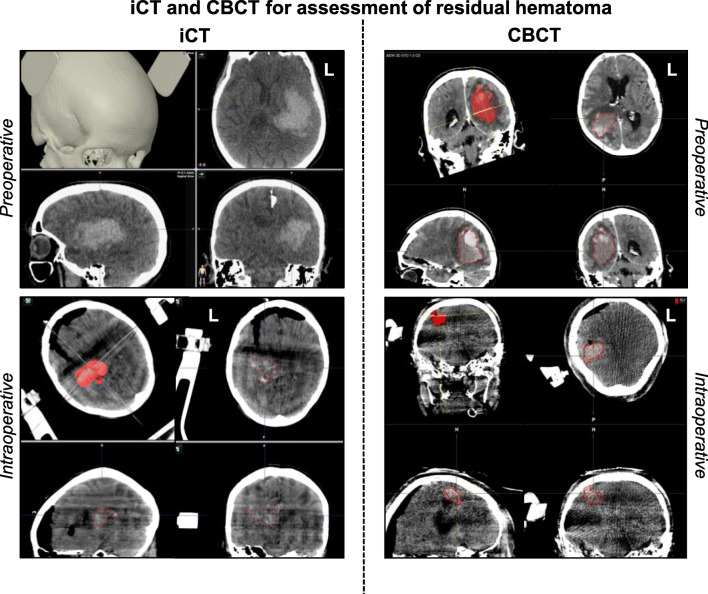
Intraoperative control imaging for hematoma detection. The preoperative CT images of patients 2 and 14 (upper panels) are displayed in comparison to intraoperative iCT and CBCT scans (lower panels) with residual hematoma for evaluation of intraoperative hematoma assessability after evacuation. L, left

1. Positioning and navigation registration
  Surgery was performed on a mobile, radiolucent, carbon fiber examination table (TRUMPF Carbon Floatline, TRUMPF Medizin Systeme GmbH & Co. KG, Saalfeld, Germany) in prone or supine position, depending on the hematoma location. The patients’ head was fixed in a radiolucent carbon fiber 3-pin head clamp (TRUMPF X-RAY, TRUMPF Medizin Systeme GmbH & Co. KG, Saalfeld, Germany). For navigated hematoma evacuation based on an intraoperatively acquired image data set, the iCT or CBCT was connected to an image guidance system with infrared tracking camera (BrainLab Curve™, Brainlab AG, Munich, Germany) and a preoperative CT scan or intraoperative CT/CBCT scan was used for automatic patient/image co-registration. The navigation reference device (Brainlab AG, Munich, Germany) was fixed to the head clamp. The navigation camera was set up to allow co-registration of the navigation reference device and the registration fiducials on the iCT gantry or CBCT flat panel detector, similar to the setup for automatic patient/image co-registration in spinal navigation [19]. The iCT or CBCT scan was executed by a CT-qualified technical radiological assistant or a surgeon qualified for performing digital volume tomography, respectively. For both imaging modalities, data sets were automatically transferred to our in-hospital Picture Archiving and Communications System (PACS). Alternatively, for navigation registration based on a preoperative image data set, manual surface matching was performed (Fig. 1).

2. Segmentation and aspiration trajectory planning
  Segmentation of the hematoma volume and planning of the aspiration trajectory was performed preoperatively or directly in the OR with image guidance software (Brainlab Cranial Planning SmartBrush Ver. 2.6.0.121 and Trajectory Ver. 2.5.1.5, Brainlab AG, Munich, Germany). After segmentation, the aspiration trajectory was planned corresponding to the longest axis of the hematoma under consideration of the transverse and sagittal planes (Fig. 2).

3. Navigated, endoscopy-assisted hematoma evacuation
  The general instruments and setup for minimally invasive hematoma evacuation are shown in Fig. 3. After navigation registration and surgical planning, the entry point and skin incision were marked and an 11-mm burr hole above the entry point was drilled in standard fashion. Following dural incision, a 19-French, single-use, neuro-endoscopy trocar (B.Braun Melsungen AG, Melsungen, Germany) with an attached navigation tracking device (Brainlab AG, Munich, Germany) was inserted into the distal third of the hematoma along the planned trajectory. A 6° video neuro-endoscope with a 2.9-mm working channel (Storz Lotta®, Karl Storz SE & Co. KG, Tuttlingen, Germany)—through which the 2.8 mm mechanical fragmentation and aspiration device is passed—was inserted through the trocar and hematoma was aspirated within the trocar sheath. Next, the endoscope was advanced into the hematoma cavity and navigated endoscopic hematoma evacuation was performed from distal to proximal under continuous irrigation. The handling of the endoscope and aspiration device was performed by one surgeon and navigation was guided by a second surgeon (4-hand technique; Fig. 3c).

4. Intraoperative control imaging
  Following ICH evacuation targeting 2/3rd of the initial hematoma volume, an iCT or CBCT control scan was performed to determine the degree of evacuation (Fig. 4). If evacuation was judged insufficient and *unrelated* to the hematoma consistency, an immediate, navigated re-aspiration attempt based on the control scan was performed.

### Statistical analysis

Descriptive summary statistics are presented as mean ± standard deviation, median, and range (minimum–maximum) or percentage, as appropriate. Statistics were calculated with GraphPad Prism for Mac (Version 8.1.0, GraphPad Software, San Diego, California, USA). For comparison of pre-, intra-, and postoperative hematoma volumes, a repeated measure one-way analysis of variance (ANOVA) with Geisser Greenhouse correction and Tukey’s multiple comparison test was performed. For comparison of the intra- and postoperative hematoma evacuation rate, a paired *t* test was performed. To compare outcome according to the GCS score, a Wilcoxon matched-pairs signed-rank test was used. All tests were two-tailed and statistical significance was set at *p* < 0.05.

## Results

### Patient characteristics and surgery

Demographic and clinical data are presented in Table 1. The median patient age was 76 years (range 42–85) with equal gender distribution and slightly predominant affection of the left hemisphere (56%) and deep location (56%). The mean preoperative hematoma volume was 54 ± 26 ml. The median ICH score at admission was 3 (range 1–4) with a preoperative GCS of 10 (range 5–14). The median time from symptom onset to surgery was 31 h (range 6–76) with a mean surgery duration of 90 ± 39 min. Sixty-seven percent of all patients had a history of platelet inhibition or anticoagulant use at the time-point of presentation and 83% received preoperative hemostasiological correction.

**Table 1:** Demographic and clinical data

No.	Age, sex	Hemisphere	Location	ICH score	OAC / PI	OAC / PI reversal	Time to surgery (hrs)	Surgery duration (min)	PreOP GCS	PostOP GCS	30-day mortality	mRS at discharge
01	71, m	Left	lobar	2	–	–	75	95	9	14	No	2
02	85, f	Left	Deep	3	PI	–	17	95	11	13	No	4
03	80, f	Left	Lobar	4	PI	–	41	134	10	14	No	3
04	78, f	Right	Lobar	2	PI	P	47	59	11	11	No	3
05	63, m	Left	Deep	3	–	–	23	147	8	12	No	5
06	64, f	Right	Deep	3	PI	P	48	65	10	14	No	5
07	72, m	Left	Deep	2	–	–	30	134	6	8	No	5
08	82, m	Right	Deep	3	NOAC	PCC	11	168	14	8	No	4
09	51, f	Left	Deep	4	–	–	76	120	6	8	No	4
10	81, f	Right	Deep	2	W	P + PCC	69	54	9	6	Yes	6
11	75, f	Right	Deep	3	NOAC	PCC	6	87	8	8	Yes	6
12	84, m	Right	Lobar	3	PI	P	6	56	12	12	No	4
13	76, m	Left	Deep	3	PI	P	8	120	10	3	Yes	6
14	58, f	Left	Lobar	2	–	–	48	69	12	15	No	2
15	79, m	Left	Lobar	1	NOAC	PCC	6	55	11	15	No	3
16	83, m	Left	Lobar	3	PI	P	31	68	13	15	No	3
17	76, f	Right	Lobar	1	NOAC	PCC	12	56	13	14	No	2
18	42, m	Right	Deep	2	–	–	42	35	5	12	No	4
	76 (42–85)			3 (1–4)	67%	83%	31 (6–76)	90 ± 39	10 (5–14)	12 (3–15)	17%	4 (2–6)

*No.*, number; *m*, male; *f*, female; *OAC*, oral anticoagulant; *PI*, platelet inhibitor; *NOAC*, non-vitamin K antagonist oral anticoagulant; *W*, warfarin; *P*, platelets; *PCC*, prothrombin complex concentrate; *GCS*, Glasgow Coma Scale; *mRS*, modified Rankin Scale score

### Intraoperative imaging and efficiency of hematoma evacuation

**Fig. 5:**
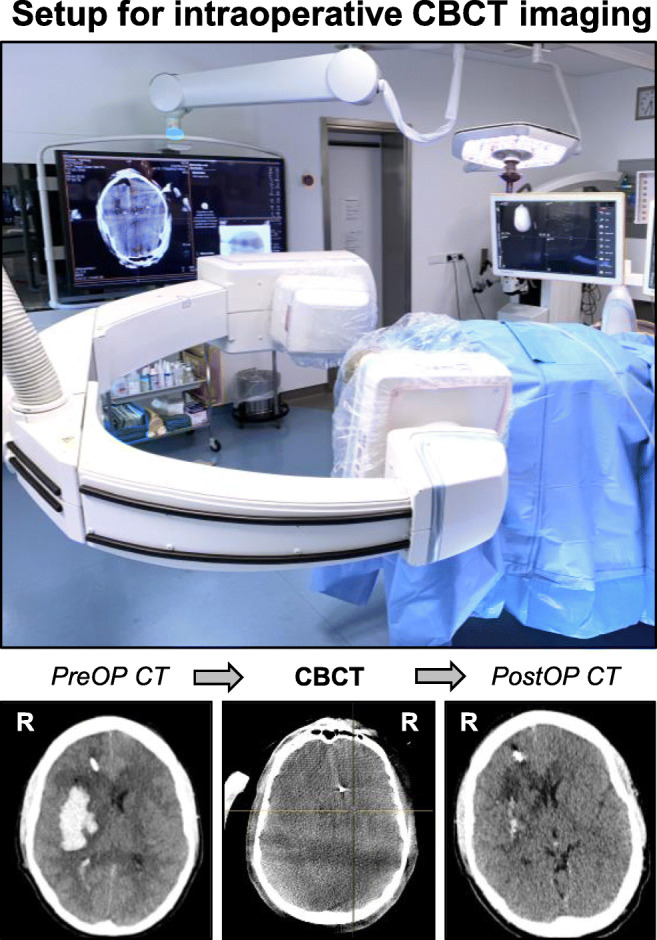
Image quality of intraoperative CBCT versus conventional CT imaging. The upper panel shows the setup for intraoperative CBCT scanning after hematoma evacuation. In contrast to iCT, the CBCT flat panel detector rotates around the patient and requires draping to maintain sterility during the scan. Below, the imaging sequence in patient 18 shows sufficient CBCT image quality for reliable intraoperative hematoma assessment compared to conventional pre- and postoperative CT imaging, despite lower signal-to-noise ratio. R, right

**Fig. 6:**
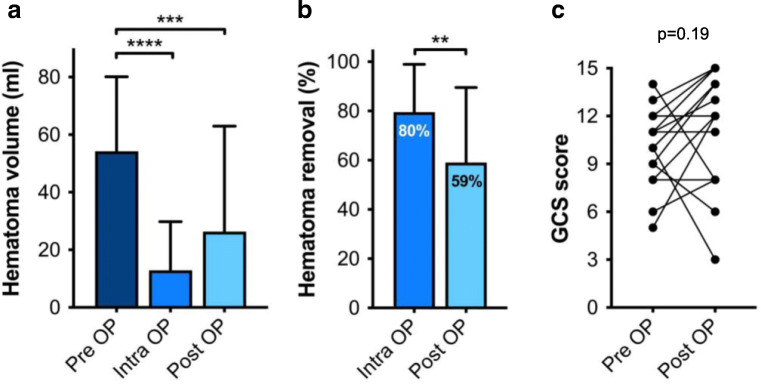
Illustration of **a** pre-, intra-, and postoperative hematoma volumes, **b** intra- and postoperative evacuation rates, and **c** pre- and postoperative Glasgow Coma Scale (GCS) score. **a** ***p < 0.001, ****p < 0.0001; repeated measures one-way ANOVA with Geisser Greenhouse correction and Tukey’s multiple comparison test. ***b*** **p < 0.01; two-tailed paired *t* test. **c** Two-tailed Wilcoxon matched-pairs signed-rank test

Data on intraoperative imaging and hematoma volume are shown in Table 2. Intraoperative imaging for hematoma removal assessment was available in 17/18 patients (94%). Intraoperative control iCT or CBCT was performed in 5/17 (29%) and 12/17 (71%) patients, respectively. Both iCT and CBCT permitted robust hematoma assessment despite a greater signal-to-noise ratio in intraoperative than in conventional CT imaging (Fig. 5). In 5/17 patients (29%), a second intraoperative re-aspiration was performed based on the iCT or CBCT control scan. In patient number 4, an iCT control scan was not possible due to logistic reasons resulting in unavailability of a radiology technician. In this patient, postoperative CT imaging revealed a hematoma evacuation rate of 43%, which prompted secondary (open) hematoma evacuation on POD 2 due to an unchanged impaired state of consciousness. In patients 8 and 13, intraoperative evacuation rates were similarly low and re-aspiration was limited by firm hematoma consistency. Here, secondary (open) hematoma evacuation was not considered due to a deep hematoma location. Despite postoperative hematoma re-expansion, the mean residual intra- (13 ± 17 ml) and postoperative (26 ± 37 ml) hematoma volumes both remained significantly lower than the preoperative (54 ± 26 ml) hematoma volume (****p < 0.0001 vs. Intra-OP and ***p < 0.001 vs. Post-OP; Fig. 6a). Accordingly, the overall intraoperative and postoperative evacuation rates were determined at 80 ± 19% and 59 ± 30%, respectively (**p < 0.01 Intra-OP vs. Post-OP; Fig. 6b).

**Table 2:** Intraoperative imaging and hematoma volume

No.	PreOP volume (ml)	IntraOP volume (ml)	Percent evacuation	PostOP volume (ml)	Percent evacuation	Device	Imaging	IntraOP control scan	IntraOP second look	Secondary revision surgery
01	31	1	97	1	97	1st gen.	iCT	Yes	No	No
02	49	10	79	37	24	1st gen.	iCT	Yes	No	No
03	53	16	70	18	66	1st gen.	iCT	Yes	No	No
04	54	N/A	N/A	31	43	1st gen.	N/A	No	No	Yes
05	43	10	78	17	62	1st gen.	iCT	Yes	No	No
06	47	6	87	36	24	1st gen.	CBCT	Yes	Yes	No
07	32	2	93	7	80	1st gen.	CBCT	Yes	No	No
08	42	30	29	27	37	1st gen.	iCT	Yes	Yes	No
09	38	1	97	4	91	1st gen.	CBCT	Yes	Yes	No
10	21	1	95	20	7	1st gen.	CBCT	Yes	No	No
11	92	8	91	21	77	1st gen.	CBCT	Yes	No	No
12	92	22	76	36	60	1st gen.	CBCT	Yes	No	No
13	125	69	44	164	0	1st gen.	CBCT	Yes	Yes	No
14	41	7	83	5	88	2nd gen.	CBCT	Yes	No	No
15	68	1	99	3	95	2nd gen.	CBCT	Yes	No	No
16	66	21	68	31	53	2nd gen.	CBCT	Yes	Yes	No
17	43	13	70	13	70	2nd gen.	CBCT	Yes	No	No
18	39	1	97	5	87	2nd gen.	CBCT	Yes	No	No
	54 ± 26	13 ± 17	80 ± 19%	26 ± 37	59 ± 30%			94%	29%	6%

*No.*, number; *N/A*, not available; 1st gen*.*, first-generation evacuation device; 2nd gen., second-generation evacuation device; *iCT*, intraoperative, computerized tomography; *CBCT*, cone-beam CT

### Outcome

A postoperative decline in the level of consciousness *despite* hematoma reduction was observed in 2/18 patients (11%). Postoperative re-hemorrhage beyond the initial hematoma volume and despite intraoperative reduction was observed in 1/18 patients (6%). Withdrawal-of-care decisions were eventually made in 3/18 patients due to a prolonged clinical course incompatible with the patients’ living will, resulting in a 30-day mortality rate of 17%. The postoperative GCS in the surviving 15 patients declined in 1 (7%), remained equal in 2 (13%) and improved in 12 patients (80%), resulting in a median postoperative GCS of 12 (range 3–15) The median postoperative mRS score at discharge was determined at 4 (2–6) (Table 1 and Fig. 6c).

## Discussion

In this feasibility study, we investigated the performance of navigated, endoscopic MIS and intraoperative 3D imaging for evacuation of supratentorial ICH through mechanical clot fragmentation and aspiration. Despite lower 3D image quality than conventional CT imaging, 3D-reconstructed cranial iCT and CBCT permitted intraoperative navigation planning and reliable evacuation rate assessment with the possibility to perform an immediate re-evacuation attempt, which is important considering the noted risk of early hematoma re-expansion.

### Patient selection, hematoma location, and timing of surgery

The current situation for ICH appears similar to the situation during the early days of thrombectomy for ischemic stroke, where patient selection was considered one of the main reasons for the initial failure of endovascular therapy, meanwhile successfully established as the standard of care [1]. In ICH, recent meta-analysis and randomized controlled trials (RCTs) have demonstrated that selection of the ideal surgical candidate depends on multiple factors, such as age, premorbid mRS, hematoma volume, clinical presentation, and timing of the procedure [16, 17, 27]. Accordingly, we only considered patients for MIS if these specific criteria were fulfilled, including availability of intraoperative imaging, stable clot volume, and exclusion of an underlying vascular pathology.

Many consider deep hematomas ideal for MIS procedures due to the large perihemorrhagic involvement of viable but highly vulnerable tissue. Although our study is underpowered to draw conclusions regarding the superiority of superficial versus deep MIS for ICH, technical success was achieved in both groups with comparable intraoperative evacuation rates of 79% and 80% but patients with superficial location appeared to benefit more considering a postoperative GCS improvement from 11 to 14 compared to patients with deep hematomas and an unchanged GCS of 9. Thus, our findings suggest that despite comparable effectiveness, MIS for deep-seated ICH should remain critically weighed.

Interestingly, time to treatment has been shown to carry weight in ischemic stroke but it has not yet been shown to play a key role in hemorrhagic stroke although patients who undergo MIS of ICH within 24 h have a 30% greater likelihood to achieve functional independence than patients who undergo MIS within 72 h [33]. Accordingly, we aimed to perform evacuation as soon as reasonably possible beyond a stable 6-h control scan. However, MIS within 24 h was only possible in 44%, due to our requirement of a stable 6-h control CT scan and the logistical availability of endoscopy, navigation, and intraoperative imaging. Further, a delay beyond 24 h was influenced by the fact that most patients were referred from external hospitals or that patients with an initial GCS of 15 experienced delayed deterioration and were only thereafter considered for MIS. On the other hand, one can argue that delayed instead of immediate evacuation could also be beneficial in clinically stable patients due to a more fluid hematoma consistency with potentially greater fragmentation and aspiration effectiveness. In our series, however, we found no association between aspiration efficiency and the timing of surgery. This suggests that aspiration effectiveness may depend on additional factors apart from time-dependent clot liquefaction, such as arachnoid involvement, for example, because hematomas that primarily dissect the brain without multiple arachnoid compartments create a circumscribed hemorrhage that better lends itself to evacuation through the sheath.

### Effectiveness of navigated, endoscopy-assisted mechanical fragmentation and aspiration

Among the various MIS modalities for ICH [18, 24, 37], we deliberately evaluated endoscopy-assisted ICH evacuation by mechanical fragmentation and aspiration, because endoscopy-assisted MIS compares favorably regarding evacuation effectiveness and functional outcome [33, 39] and remains the most widely investigated MIS for ICH technology [2, 15, 21, 28, 37, 39]. Although stereotactic thrombolysis has the benefit of simplicity, low cost, and ease of use, it remains limited by a delayed hematoma evacuation and up to 40% risk of suboptimal or poor catheter placement accuracy, which may limit effectiveness and promote secondary neurological injury [32]. Further, delayed ICH resolution may prolong recovery with an increased risk of systemic complications that can suggest poor long-term neurological outcome and trigger premature withdrawal of care decisions [6, 30]. In contrast, a navigated, endoscopy-assisted MIS approach has the advantage of immediacy, precision, and direct visibility with the chance to address active bleeding through coagulation or irrigation. As a further technical advancement, the utilization of atraumatic mechanical fragmentation and aspiration instead of endoscopic aspiration alone appears feasible, effective, and safe [15, 21, 37, 38], which is supported by the finding that none of our patients required conversion to an open craniotomy.

For the first time, MISTIE III evidenced that reducing the absolute hematoma volume below a specific threshold appears to be the most beneficial therapeutic target [3]. Still, this pre-defined 15-ml threshold was only reached in 60% of all MIS patients in MISTIE III. In the present pilot study, mechanical fragmentation and aspiration compared favorably to these results with an overall intraoperative evacuation rate of 80%, mean residual hematoma volume of 13 ml and 71% of all patients below the 15-ml threshold. Although this effect was somewhat alleviated by hematoma re-expansion between the time-point of intraoperative imaging and the postoperative control CT on POD 1, the adjusted evacuation rate of 59% still compares well to the initial multicenter experience of other groups using mechanical fragmentation and aspiration for endoscopic ICH evacuation [37]. In our series, this may have been caused by residual bleeding that was not addressed by coagulation or sufficient irrigation but active bleeding from injured (micro) vessels was visually excluded in each case by using the previously described underwater blood aspiration technique (SCUBA) [21]. Alternatively, non-specific bleeding, blood pressure, and coagulation management may be responsible for this effect. Nevertheless, the generalizability of our findings need to be confirmed by ongoing RCTs and other neurosurgical groups, considering the higher cost and greater challenges that we noted in regard to logistics, setup, workflow, and technical skill, which fall in line with other MIS techniques [25, 31].

### Intraoperative CT and CBCT imaging for ICH evacuation assessment

Surgical performance in MIS for ICH is crucial to ensure the best possible chances of favorable recovery [3]. This is particularly important in endoscopic clot fragmentation and aspiration, where intraoperative performance cannot be evaluated as clearly as in an open craniotomy and mirrored by the reported evacuation effectiveness ranging between 54% [37] and 88% [21], similar to our present experience. For detection of ICH, CT remains the gold standard due to its comparatively low cost, wide availability, fast acquisition, clear visualization, and simple interpretation. Although previous studies in the field of stereotactic and tumor surgery have shown feasibility and benefit of using iCT and CBCT for direct control of electrode positioning [8, 9, 23, 36] and extent of tumor resection [4, 5], information on the feasibility of iCT or CBCT for identification of ICH does not exist. In the present study, ICH assessment with ICT and CBCT was feasible with both technologies, despite an image quality advantage of iCT over CBCT. Most importantly, both imaging modalities provided enough information to prompt an immediate re-aspiration attempt in nearly 1/3rd of our cohort, which underlines the importance of routinely implementing direct intraoperative evacuation assessment in every MIS case considering that the single patient who did not receive iCT control scanning required secondary open surgery. Regarding workflow and applicability, iCT remained hampered because scan execution required a qualified radiologist or radiology technician, whereas CBCT was utilized 24 h a day by a qualified surgeon alone. Moreover, the CBCT user interface permitted a completely independent operation without the assistance of circulating OR personnel, which made CBCT particularly attractive in settings when radiological assistance or specialized OR personnel was not available.

### Limitations

Although our study inherently lacks power due to its retrospective nature, small sample size, lack of a control group and long-term follow-up, the investigated patient cohort is representative of ICH patients considered for MIS in regard to clinical presentation, demographics, hematoma volume, hematoma location, and ICH grade. Another limitation is that we did not analyze radiation exposure. Importantly, the surgeon must consider that intraoperative iCT and CBCT imaging exposes the patient to at least one additional iCT or CBCT scan for intraoperative evacuation assessment. On the other hand, we believe that the additional iCT or CBCT radiation exposure is justifiable in MIS for ICH considering the severity of the disease and impact of targeting a critical volume threshold. Although radiation exposure in iCT imaging is most likely higher than in CBCT imaging, it has been demonstrated that the effective iCT patient dose remains at an acceptable level compared to CBCT technology [14, 34]. Nevertheless, the goal must be to minimize radiation exposure as much as possible. In the future, this could be accomplished by eliminating a routine preoperative 6-h control CT in clinically stable patients, given the potential benefit of ultra-early MIS evacuation [33]. Regarding postoperative imaging, our findings suggest to currently maintain routine imaging in order to gage procedural success on the intraoperatively achieved target volume. Most importantly, our setup ensured complete elimination of radiation exposure of the OR team, because iCT and CBCT were remotely executed without personnel remaining in the OR during the scan. Considering the cumulative dose that the OR personnel is exposed to during their career this may be of even greater health-related relevance than the individual patient-associated dose, which is outweighed by the potential advantages that iCT and CBCT may have to offer in regard to performance assessment, safety, patient outcome, and reduction of surgical revision rates. In conclusion, MIS for ICH with mechanical fragmentation and aspiration represented a feasible and safe treatment for a patient population with very limited medical and surgical options.

## Electronic supplementary material

ESM 1 (PDF 28 kb)
