# Supplementary material for: Intraoperative CT and cone-beam CT imaging for minimally invasive evacuation of spontaneous intracerebral hemorrhage
Source: Acta Neurochir (Wien). 2020 Mar 19;162(12):3167–77. doi: 10.1007/s00701-020-04284-y (PMC7593312; doi:10.1007/s00701-020-04284-y)
Supplement: Supplementary file 1 — (PDF 28 kb) [file 701_2020_4284_MOESM1_ESM.pdf]

Online Supplement

Intraoperative CT and cone-beam CT imaging for minimally invasive evacuation of spontaneous intracerebral hemorrhage

[1]Nils Hecht, MD; [1]Marcus Czabanka, MD; [1]Paul Kendlbacher, MD;

[1]Julia-Helene Raff; [2]Georg Bohner, MD; [1]Peter Vajkoczy, MD

[1]Department of Neurosurgery and Center for Stroke Research Berlin (CSB),

[2]Department of Neuroradiology,

Charité - Universitätsmedizin Berlin, Berlin, Germany

Patient management

During surgery, patients were anesthetized with propofol and remifentanil. Heart rate, arterial blood pressure, peripheral oxygen saturation and body temperature were monitored continuously. The intraoperative mean arterial pressure (MAP) was targeted at 70 mmHg. Arterial blood gases were sampled every 30 minutes and the end-expiratory carbon dioxide concentration was maintained at a level corresponding to an arterial partial pressure of CO_2 between 38 and 42 mmHg. After surgery, patients were transferred to the intensive care unit. In patients with a postoperative GCS $\leq$ 8, intracranial pressure (ICP) was monitored and patients remained intubated and sedated until ICP was within normal ranges. A critical ICP threshold was defined as an ICP > 20mmHg for a period longer than 10 minutes and treated according to national and international guidelines with cerebrospinal fluid drainage, osmotic therapy and deep sedation. Blood gases, electrolytes and glucose were controlled every 4 hours. In all patients, a systolic blood pressure below 140mmHg was targeted.

Intraoperative imaging

Intraoperative CT or CBCT imaging was performed with the mobile AIRO® iCT (Brainlab AG, Munich, Germany) or robotic Artis Zeego® II CBCT (Siemens Healthcare, Forchheim, Germany). The mobile iCT is designed to function within an existing OR suite and contains a mobile CT gantry (diameter 107cm; dimensions 30.5cm x 38cm), which houses the X-ray tube, 32 slice helical scan detector array, high-voltage generator, air cooling system and battery pack. The stationary, robotic CBCT uses a 30cm x 40cm flat panel detector C-arm to obtain fluoroscopic images at submillimeter spatial resolution, which are immediately postprocessed into a 3D data set.
